# Characterization of DicB by partially masking its potent inhibitory activity of cell division

**DOI:** 10.1098/rsob.160082

**Published:** 2016-07-27

**Authors:** Shaoyuan Yang, Hairun Pei, Xiaoying Zhang, Qiang Wei, Jia Zhu, Jimin Zheng, Zongchao Jia

**Affiliations:** 1 College of Chemistry, Beijing Normal University, Beijing 100875, People's Republic of China; 2 Department of Biomedical and Molecular Sciences, Queen's University, Ontario, CanadaK7L 3N6

**Keywords:** prophage, MBP, DicB, cell division inhibition, MinC

## Abstract

DicB, a protein encoded by the Kim (Qin) prophage in *Escherichia coli*, inhibits cell division through interaction with MinC. Thus far, characterization of DicB has been severely hampered owing to its potent activity which ceases cell division and leads to cell death. In this work, through fusing maltose-binding protein to the N-terminus of DicB (MBP–DicB), we successfully expressed and purified recombinant DicB that enabled *in vitro* analysis for the first time. More importantly, taking advantage of the reduced inhibitory activity of MBP–DicB, we were able to study its effects on cell growth and morphology. Inhibition of cell growth by MBP–DicB was systematically evaluated using various DicB constructs, and their corresponding effects on cell morphology were also investigated. Our results revealed that the N-terminal segment of DicB plays an essential functional role, in contrast to its C-terminal tail. The N-terminus of DicB is of critical importance as even the first amino acid (following the initial Met) could not be removed, although it could be mutated. This study provides the first glimpse of the molecular determinants underlying DicB's function.

## 1. Introduction

Prophages are defined as those genomes that can be integrated into a bacterial genome or form extrachromosomally. The introduction of phage DNA into the bacterial host can result in a beneficial phenotype and help cope with competitive environments [1–4]. The most intensively studied effect of prophages is superinfection exclusion systems, which inhibit other phages including themselves in both Gram-negative and Gram-positive organisms [5]. They produce proteins to block phage replication by altering components of the cell envelope. Resistance to prophage superinfection not only helps a bacterial host fight against its environment by fending off phage infection, but also enhances bacterial virulence [6,7]. Some prophage-encoded genes are relied on by bacterial pathogens for toxin production, such as cytotoxin from *Pseudomonas aeruginosa* [8], diphtheria toxin from *Corynebacterium diphtheria* [9] and neurotoxin from *Clostridium botulinum* [10]. Yet, prophage-encoded toxins are not always pernicious. The gamma-proteobacteria symbiont *Hamiltonella defensa* protects its aphid host from attack by a parasitoid wasp depending on an *H. defensa* prophage-encoded toxin [11]. Although study of prophage has been ongoing for decades, the role of many prophage factors has remained largely unclear.

Owing to various genome rearrangements and gradual decay, parts of prophages may be trapped in the chromosome of the host and become inactive in cell lysis, phage particle production and plaque formation, which are referred to as cryptic prophages [1]. However, many examples indicate functional roles of prophages in cell physiol­ogy. In *Escherichia coli*, the genes of prophages make important contributions to host survival in adverse environmental conditions [12]. Gene *dicB* is part of the *dic* operon, which resides in cryptic prophage Kim (Qin), one of nine cryptic prophages in the *E. coli* K-12 strain [13]. The expression of DicB is repressed under normal conditions. FtsZ is the major cytoskeletal protein in the bacterial cytokinesis machine, which forms a filamentous ring (the Z ring) at the centre of the cell [14–16]. It is well established that cell division is spatially regulated by Min oscillation, including participation of proteins MinC, MinD and MinE, through inhibition of Z ring formation [17]. Nevertheless, *in vivo* study revealed that the division-inhibitory activity of MinC can also be enhanced by DicB. This inhibition is not spatially regulated by MinE [17–19]. Through microscopy experiments, Johnson *et al.* [20] revealed that DicB competes with MinD for complex formation with MinC. de Boer *et al.* [21] concluded that inhibition of cell division by DicB is dependent on expression of MinC. DicB is found to interact directly with the C-terminal domain of MinC and the MinC/DicB complex is shown to have high affinity for septal ring structures *in vivo* [20]. When DicB is expressed, cell division can be rapidly ceased [22]. Thus far, owing to its potent activity that leads to cell death through cell division inhibition, characterization of DicB has, however, been restricted to *in vivo* experiments and has hence been severely limited. A better understanding of DicB's biochemical function will not only provide novel insights into MinC/DicB regulation of cell division but also add important information about the function of prophage-encoded factors.

In this work, through fusing maltose-binding protein to the N-terminus of DicB (MBP–DicB), we successfully expressed and purified recombinant DicB that enabled *in vitro* analysis for the first time. Our pull-down assay demonstrated direct interaction between MBP–DicB and MinC *in vitro*. Furthermore, taking advantage of reduced inhibitory activity of MBP–DicB, we were able to study its effects on cell growth and morphology. Inhibition of cell growth by MBP–DicB was systematically evaluated, and the effects of these constructs on cell morphology were observed for various DicB derivatives. We revealed that the N-terminal segment of DicB plays a more critical functional role than does its C-terminal tail. The N-terminus of DicB is critically important for DicB's function as even the first amino acid (following the initial Met) could not be removed, albeit it could be mutated. This study provides the first glimpse of the molecular determinants underlying DicB's function.

## 2. Results

### 2.1. Overexpression of MBP–DicB and *in vitro* pull-down experiment

We first attempted to express DicB using various vectors containing the full-length *dicB* gene in BL21(DE3) cells. These included pET-22b-DicB, pET-32a-DicB and pET-28b-DicB. Even in the absence of isopropyl β-d-1-thiogalactopyranoside (IPTG) induction, all DicB transformants were unable to grow clones on plates (table 1). We speculate that a very small amount of basal expression of DicB in BL21(DE3) cells was sufficiently potent to inhibit cell division and result in cell death on plates.

**Table 1.** Cell division inhibition tests of DicB constructs.

DicB full-length	growth on LB plates	DicB-truncations	growth on LB plates	DicB-mutants	growth on LB plates
pET-22b-DicB	no	DicB-ΔN2^a^	yes		
		DicB-ΔN3	yes	DicB-K2A	no
		DicB-ΔN4	yes	DicB-K2G	no
		DicB-ΔN5	yes	DicB-K2R	no
		DicB-ΔN6	yes		
		DicB-ΔN7	yes	DicB-T4A	no
pET-32a-DicB	no	DicB-ΔN13	yes	DicB-L5A	yes
		DicB-ΔN26	yes	DicB-K2A/T3A^b^	no
		DicB-N26	yes	DicB-T4A/L5A	yes
		DicB-ΔN26	yes		
		DicB-ΔC8	no	DicB-K2A/T3A/T4A/L5A	
pET-28b-DicB	no	DicB-ΔC12	yes		yes
		DicB-ΔC16	yes		
		DicB-ΔC20	yes		

^a^DicB-ΔN2 refers to the construct in which the N-terminal two amino acids of DicB were truncated.

^b^DicB-K2A/T3A refers to the construct in which K2 and T3 were both mutated to alanine.

**Figure 1.**
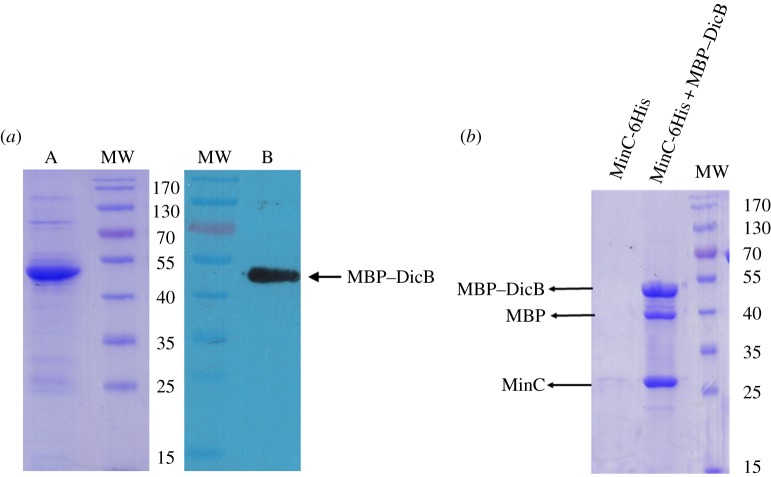
MBP–DicB purification and its interaction with MinC. (*a*) SDS–PAGE analysis of MBP–DicB protein purified by Ni-NTA column from bacterial lysate. MW, molecular weight marker; lane A: eluted protein fraction from His-Trap HP column; lane B: western blotting analysis of the duplicated gel transferred onto PVDF membrane and probed with a mouse monoclonal anti-His-HRP-conjugated antibody. Sample loaded onto lane A was five times of that on lane B. (*b*) Pull-down experiment of MBP–DicB and MinC. The control experiment was done using the same procedures without MBP–DicB in order to confirm MinC has no non-specific binding to the amylose column. MBP–DicB readily pulled down MinC that contains no MBP tag. The observed MBP band is a result of partial degradation of MBP–DicB.

We next fused maltose-binding protein (MBP) to the N-terminus of DicB, based on the fact that MBP has been recognized as one of the most effective solubilizing agents and frequently observed to help express toxic proteins [23,24]. The MBP–DicB transformant enabled clones to grow on plates. As a result, we were able to carry out DicB overexpression and purification, and obtained a large amount of pure recombinant MBP–DicB protein (figure 1*a*). This is the first time, to the best of our knowledge, that recombinant DicB has been successfully produced, which has enabled us to carry out DicB *in vitro* characterization, not possible before. Using the recombinant DicB sample, we performed pull-down assay. As shown in figure 1*b* and the electronic supplementary material, figure S1, there is unambiguous interaction between MBP–DicB and MinC, providing direct evidence of their interaction which is consistent with cell biology experiments [20].

### 2.2. Cell division inhibition of MBP–DicB

**Figure 2.**
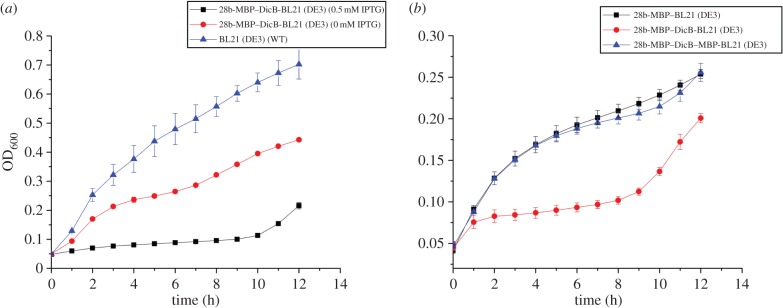
Effects of MBP–DicB on cell growth. (*a*) Growth curves of WT *E. coli* BL21(DE3) (blue) and MBP–DicB strain with (black) and without (red) IPTG induction under aerobic conditions at 37°C. (*b*) Growth curves of MBP (black), MBP–DicB (red) and MBP–DicB–MBP (blue) strains with 0.1 mM IPTG induction under aerobic conditions at 37°C. All experiments were completed in triplicate and performed twice. The standard error of the mean was used to calculate the error bars. For those points where experimental variations are too small, their error bars are not visible.

To test whether MBP–DicB still retains its role in cell division inhibition, we systematically investigated growth of *E. coli* BL21(DE3) strain containing pET-28b-MBP–DicB in comparison with wild-type (WT; figure 2*a*). We found even without IPTG, growth of MBP–DicB strains decreased compared with WT. After IPTG induction, growth of MBP–DicB strain significantly reduced to less than a half of WT, indicating expression of MBP–DicB strongly inhibited cell growth of *E. coli*. We speculate that although MBP masks certain activity of DicB, MBP–DicB can still inhibit cell division. However, when MBP was fused to both N- and C-termini of DicB, MBP–DicB–MBP strains grew faster than MBP–DicB and were similar to MBP expression strains, suggesting that the two MBP molecules almost completely masked the activity of DicB (figure 2*b*).

**Figure 3.**
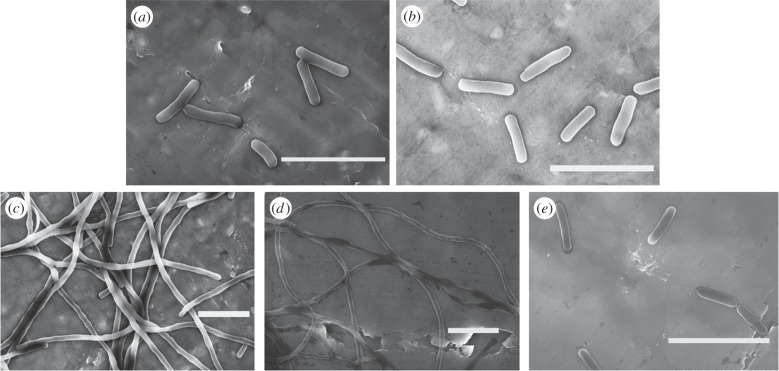
Scanning electron micrographs of WT, MBP–DicB and MBP–DicB–MBP expression in *E. coli* BL21(DE3) strains. (*a*) WT. (*b*) Negative control: MBP + 0.5 mM IPTG. (*c*) MBP–DicB + 0 mM IPTG. (*d*) MBP–DicB + 0.5 mM IPTG. (*e*) MBP–DicB–MBP + 0.5 mM IPTG. Scale bars, 5 µm.

To test whether MBP–DicB affects cell morphology, we next carried out scanning electron microscopy (SEM) experiments. As shown in the SEM images (figure 3), MBP–DicB expression strain was significantly elongated. Cells dimension measurements indicated MBP–DicB expression strain was more than 50 times longer than WT strain (greater than 100 µm versus 2.6 µm). Furthermore, MBP–DicB strain without IPTG induction also exhibited very long cells (figure 3*d*), confirming our earlier observation that there was basal expression in *E. coli* BL21(DE3) which resulted in cell division inhibition. In comparison, MBP–DicB–MBP strain displayed normal cell length (figure 3*e*), consistent with the cell growth results (figure 2).

### 2.3. The N-terminus of DicB is essential for function

Interestingly, whereas fusion of MBP or enhanced green fluorescent protein (EGFP) to the N-terminus of DicB did not result in cell death, when MBP or EGFP was fused to the C-terminus of DicB, these transformants disabled growing clones on plates similar to full-length DicB constructs (electronic supplementary material, table S1). It implies that the N-terminus of DicB plays an essential functional role in contrast to its C-terminal tail. Based on the predicted secondary structure of DicB (electronic supplementary material, figure S2), we prepared various DicB constructs and tested their effects (table 1). We found that when the C-terminal eight amino acids of DicB were removed, no colony grew on plates. In contrast, once the N-terminal amino acids of DicB were removed (various truncations ranging from 2 to 26 aa), all of these transformants were able to grow clones on plates (table 1). These observations indicate that the N-terminus is more critical than the C-terminal tail (8 aa) for DicB activity. Even more strikingly, the removal of a single residue, the first Lys following the initiating residue (Met), diminished the cell inhibition activity of DicB.

**Figure 4.**
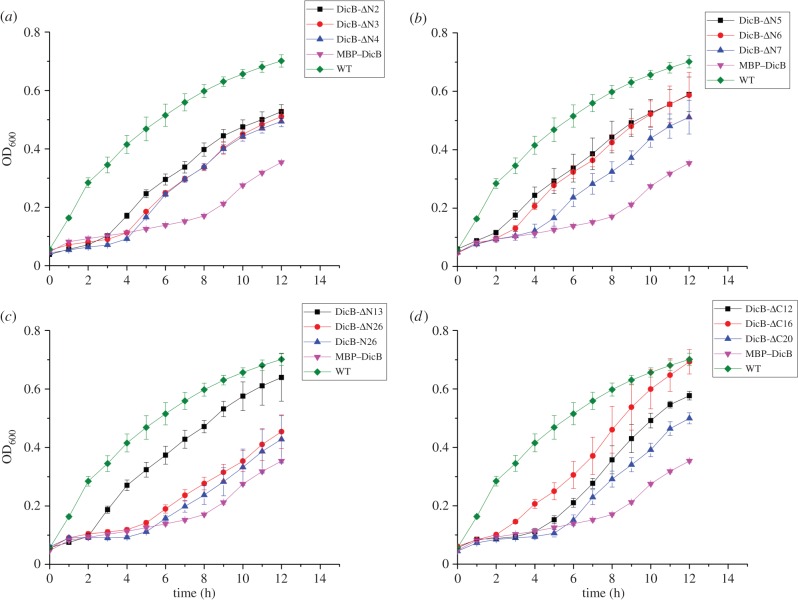
Growth assay of DicB truncation strains. Growth assay for WT *E. coli* BL21(DE3) (green), MBP–DicB strain (pink) and different truncations of DicB strains (table 1) under aerobic conditions at 37°C. All experiments were completed in triplicate and performed twice. The standard error of the mean was used to calculate the error bars. For those points where experimental variations are too small, their error bars are not visible.

**Figure 5.**
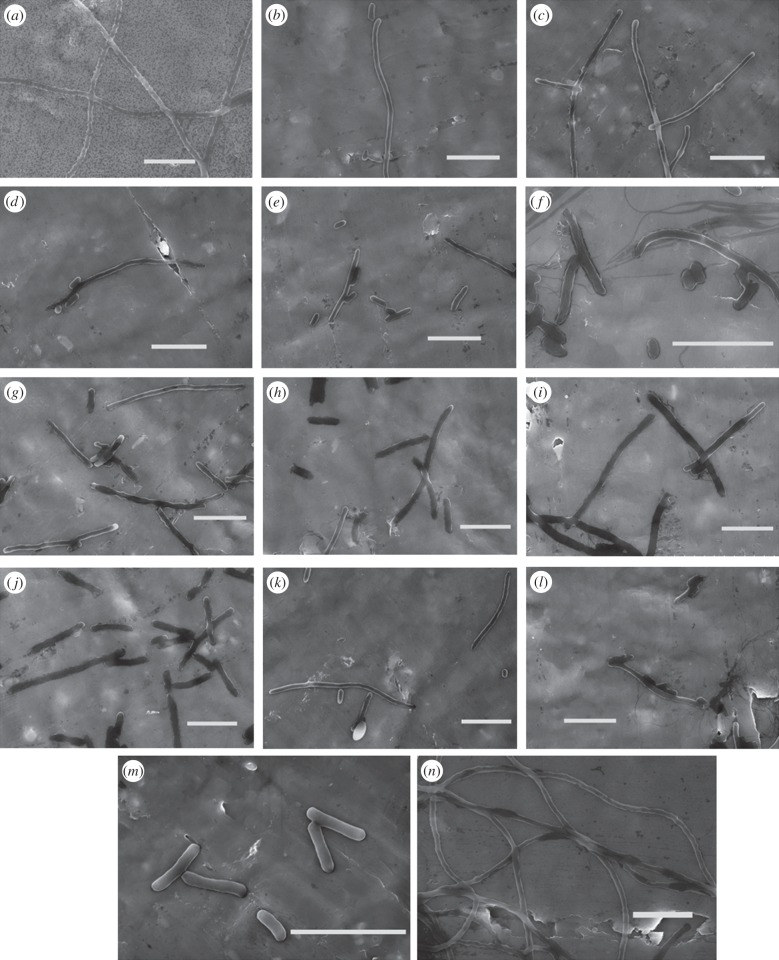
Scanning electron micrographs of different truncation derivatives of DicB (*a*–*l*) and MBP–DicB (*n*) expression in *E. coli* BL21(DE3). (*a*) DicB-ΔN2; (*b*) DicB-ΔN3; (*c*) DicB-ΔN4; (*d*) DicB-ΔN5; (*e*) DicB-ΔN6; (*f*) DicB-ΔN7; (*g*) DicB-ΔN13; (*h*) DicB-ΔN26; (*i*) DicB-N26; (*j*) DicB-ΔC12; (*k*) DicB-ΔC16; (*l*) DicB-ΔC20; (*m*) *E. coli* BL21(DE3); (*n*) MBP–DicB. Cells of DicB truncation derivatives were generally much longer than WT but shorter than MBP–DicB, which verified that the inhibitory activity of DicB truncations were weakened but not completely abolished. Scale bars, 5 µm.

To quantitatively assess the inhibitory activity of these 12 DicB constructs (table 1), we performed cell growth assay using WT and various DicB truncation strains. Growth curves (figure 4) showed that the growth of MBP–DicB overexpression strain was the slowest. The other 12 DicB constructs grew faster than MBP–DicB strain but slower than WT, demonstrating that their inhibitory activity was less than MBP–DicB but still existed. Our LB plate results confirmed that colonies of these DicB truncation strains were fewer than WT and more than MBP–DicB (electronic supplementary material, figure S3). Western blotting showed that these DicB derivatives were expressed at almost similar levels (electronic supplementary material, figure S4). Consistent with the growth curves, SEM images (figure 5) showed almost all the strains of the 12 DicB constructs displayed much shorter length than MBP–DicB. We speculate that the N-terminal 13 amino acids function together to enable its inhibitory activity. To investigate interaction between these DicB truncations and MinC, we performed pull-down experiments, using MBP-fused DicB truncations. There was still interaction between these DicB derivatives and MinC (electronic supplementary material, figure S5*a*). N-terminal truncations with less residues removed (ΔN6 and ΔN7) showed stronger interaction with MinC than those with more residues removed (ΔN13, ΔN26 and N26). These results indicate that the N-terminal 14 amino acid residues are probably important for interaction with MinC and thus play a key role in DicB's function.

### 2.4. The N-terminal integrity is important for DicB's function

Considering the critical importance of the N-terminal Lys residue that could not be removed, we sought to probe whether mutation would affect DicB's activity, using site-directed mutagenesis. To this end, we generated three mutants, namely K2A, K2G and K2R. The first two were intended to drastically reduce the size and remove the positive charge of Lys. The third one was designed as a conservative mutation. Unexpectedly, all three DicB-K2 mutants retained strong inhibitory activity and inhibited clone growing on plates (table 1). These results strongly suggest that the second residue at the lysine position is essential, whereas the type of amino acid does not seem to be important. In order to learn more about the N-terminus of DicB, we conducted a series of additional mutations in the first five amino acids of DicB's N-terminus. Our results showed that mutation at the fifth leucine weakened the activity of DicB and allowed clone formation on plates but mutations at the third and fourth amino acids maintained DicB's activity (table 1).

### 2.5. Overexpression of FtsZ indirectly suppressed the inhibitory activity of DicB

Considering the study of de Boer *et al.* [21] revealing that the MinC/DicB division inhibition mechanism can be reversed by high levels of the *ftsZ* gene product, we carried out cell growth assay and SEM experiments using strains expressing MBP–DicB with FtsZ. As seen in the electronic supplementary material, figure S6, growth of MBP–DicB/FtsZ strain was faster than MBP–DicB strain, but still slower than FtsZ strain. Moreover, SEM images (electronic supplementary material, figure S7) showed that cell length of MBP–DicB/FtsZ strain was much shorter than MBP–DicB strain and approximately three times as long as WT (7–8 µm versus 2.6 µm). Intriguingly, cell morphology of MBP–DicB/FtsZ was like strings of beads. Similar to MBP–DicB, expression of MinC led to very long cell (greater than 30 µm; electronic supplementary material, figure S7*b*), and the co-expression of MinC with FtsZ (electronic supplementary material, figure S7*d*) also caused cell morphology into strings of beads of much shorter length.

In order to determine whether FtsZ directly or indirectly interferes with the inhibitory activity of DicB, we performed pull-down assay between MBP–DicB and FtsZ. As seen in the electronic supplementary material, figure S8, there is no detectable interaction between MBP–DicB and FtsZ *in vitro*, suggesting that expression of FtsZ suppressed the inhibitory activity of DicB indirectly.

## 3. Discussion

Recently, an increasing level of research activity has focused on cell division factors encoded by phages, including a T7 gene product interacting with FtsZ [25] and a λ prophage-encoded factor Kil interacting with FtsZ and ZipA to prevent FtsZ assembly into a division-competent ring structure [26]. A peptide encoded by lytic SPO1 phage of *Bacillus subtilis* inhibits host cell division prior to lysis [27]. Therefore, a variety of prophage-encoding factors function in cell division. However, detailed mechanisms by which phage-encoded factors inhibit bacterial cell division remain to be elucidated. DicB, a protein encoded by Kim (Qin) prophage, inhibits cell division through interaction with MinC [21]. Understanding interaction between DicB and MinC is important in studying cell division inhibition. Thus far, nearly all research on DicB has been limited to *in vivo* study owing to its potent activity that inhibits cell division and leads to cell death. With the help of MBP, we have successfully expressed and purified recombinant DicB, which has enabled *in vitro* analysis for the first time. Pull-down assay showed that MBP–DicB interacts with MinC directly *in vitro* (figure 1), consistent with the yeast two-hybrid experiments and microscopy observations [20].

It is widely accepted that Min proteins undergo an oscillation in ensuring cell division occurring at mid-cell in rod-shaped bacteria. However, little is known about how products encoded by bacteriophage may inhibit cell division. In this study, we revealed that the N-terminus of DicB is of critical importance and not even the first amino acid (following the initial Met) can be removed. We found that MBP or other protein (e.g. EGFP; electronic supplementary material, table S1), when fused to the N-terminus of DicB, can partially mask the potent inhibitory activity of DicB. As a result, MBP–DicB permitted colony formation on plates. In contrast, C-terminal fusion (DicB–MBP) strain still disabled clone growing. Taking our cell division inhibition tests, cell growth experiments and SEM images of various DicB constructs together, we conclude that the N-terminus is essential, whereas the C-terminal tail (8 amino acids) is not as important. However, it does not mean that the C-terminus lacks function. DicBΔC12, DicBΔC16 and DicBΔC20 mutants exhibited short cells, suggesting that the residues beyond the last eight amino acids participate in inhibitory activity. Interestingly, MBP–DicB–MBP construct displayed similar cell length to WT and its growth curve was close to MBP strain. The importance of N-terminus was further substantiated by truncation studies. Unexpectedly, even the first amino acid lysine (following the initial Met) could not be removed, although it could be replaced by other amino acids. This result demonstrates the residue at the second lysine position is essential but the type of amino acid is not critical. Moreover, the fifth leucine in the N-terminus is also critical as its mutation drastically influenced DicB's function. However, the structural basis underlying these effects is unclear and needs to be further investigated.

MinC has been demonstrated to interact with DicB *in vivo* [28] and *in vitro* (this work). The N-terminal domain of MinC interacts with FtsZ and interferes FtsZ's ability to form the Z-ring [29,30]. The C-terminal domain of MinC forms a constitutive dimer and interacts with MinD as well as FtsZ. Ghosal *et al.* discovered that MinC and MinD together form a new class of nucleotide-dependent, alternating copolymeric filaments [18]. They suggest that membrane-bound MinCD copolymers constitute the active inhibitor complex that spatially regulates Z-ring assembly [18]. Therefore, MinC inhibits the division at all of the potential division sites but normally requires the activity of MinD for its function [31]. MinE promotes mid-cell division by excluding MinCD from the mid-cell site. The fact that overexpression of MinC leads to cell filaments is because of the inability to form proper septa at the middle of the cell.

As evidenced by our SEM and cell growth assays, the activity of DicB was partially masked by MBP. Even so, MBP–DicB still retained strong activity of cell division inhibition. However, cell division could be interrupted by overexpression of FtsZ indirectly. Unexpectedly, MBP–DicB–MBP overexpression strain exhibited similar cell morphology to WT, indicating the activity of DicB was masked almost completely.

DicB is known to compete with MinD for complex formation with MinC [20,28]. Through yeast two-hybrid assay, Johnson *et al.* [20] observed a stronger interaction between MinC and DicB than that between MinC and MinD. Moreover, Takashi *et al.* [32] observed direct interaction of MinC with monomeric FtsZ in solution by fluorescence resonance energy transfer. Based on our findings and literature, we propose a functional mechanism of DicB, MinC and FtsZ in cell division control. In the WT cell, FtsZ filaments form at the mid-cell site as a result of the spatial regulation via Min oscillation. We suggest that when expression of DicB *in vivo* reaches certain levels, its interaction with MinC would sufficiently compete with MinD. As a result, MinC would dissociate from MinD and be released from the membrane, leading to an increasing amount of MinC in the cytoplasm. This excessive amount of MinC interacts directly with monomeric FtsZ, leading to the cell being unable to form FtsZ filaments and thus unable to form proper septa at the middle of the cell, which abrogates cell division and growth. Nevertheless, when FtsZ is co-expressed with DicB, the Z ring structure could be formed because of a sufficient amount of FtsZ in cytoplasm, resulting in appropriate cell division. According to the findings of Johnson *et al.* [33], there is direct interaction between ZipA and ^D^MinC/DicB complex *in vivo*, and ZipA is required for recruitment of the ^D^MinC/DicB complex to FtsZ rings. Moreover, ZipA has been demonstrated to interact directly with polymeric FtsZ [34,35]. We propose that ZipA plays a role in the ‘connecting bridge’ that links the MinC/DicB complex with FtsZ and helps to activate the inhibitory activity of MinC/DicB.

When the N-terminus of DicB is fused with MBP, a 40 kDa protein, MBP–DicB is able to partially compete with MinD and interact with MinC (electronic supplementary material, figure S9). Consequently, MBP–DicB overexpression can, to a lesser extent, result in the cell's inability of septa formation and inhibit cell division. When MBP was fused to both C- and N-termini of DicB, the interaction between DicB and MinC was essentially abolished, and thus unable to interrupt the recruitment of MinC to the membrane by MinD (electronic supplementary material, figure S5*b*). Therefore, the morphology of MBP–DicB–MBP overexpression strain was similar to WT.

In summary, our study provides the first glimpse of the molecular determinants underlying DicB's function, enabled by the successful production of recombinant DicB through MBP fusion, which otherwise would not been possible owing to DicB's toxicity. Taking advantage of the reduced inhibitory activity of MBP–DicB, we were able to carry out *in vitro* analysis for the first time. We found that MBP–DicB possesses strong activity of cell division inhibition as evidenced by cell growth assays and SEM images. We also revealed that the N-terminus of DicB plays an essential functional role, because even the first amino acid (following the initial Met) could not be removed, although it could be mutated. Moreover, the fifth leucine is of critical importance as it could not be mutated. Understanding the structural basis of interaction between DicB and MinC, as well as between MinC and FtsZ, is critically important. Our result showing direct interaction of DicB and MinC *in vitro* for the first time establishes the basis for further structural characterization. Additionally, regulation of cell division inhibition in the context of the remarkable properties of DicB also remains to be investigated.

## 4. Material and methods

### 4.1. Strains and growth conditions

Standard genetic methods including transformation were used for strain construction. Bacterial strain TOP10 was used for general cloning and plasmid maintenance. BL21(DE3) strain was used for protein expression, pull-down assay, SEM experiments and cell division inhibition test experiments. Cells were grown in Luria–Bertani (LB) medium at 37°C. Except for cell growth assay, optical density readings at 600 nm (OD_600_) were measured in a 1 cm optical path length using a Powerwave XS Microplate spectrophotometer (Bio-Tek Instruments, Inc.). LB medium was supplemented with ampicillin (100 µg ml^−1^; Amressco), kanamycin (100 µg ml^−1^; Caisson) as needed. Gene expression from vectors derived from pET28- and pET22- (Novagen-EMD Millipore) was induced with 0.5 mM IPTG (Amressco).

### 4.2. Plasmids construction

The plasmids used in this study are listed in the electronic supplementary material, table S2. Genes encoding MBP–DicB were inserted into a modified pET-28b vector (mod-pET28b) using standard cloning strategies. The N-terminal His-tag in the original pET-28b vector was replaced by an MBP-tag and a cleavage site for Precision protease to create mod-pET28b. After insertion, DicB was expressed with MBP at its N-terminus and His-tag at its C-terminus. In the construct of MBP–DicB lacking C-terminal His-tag, the termination code (TGA) was inserted after MBP–DicB. For MBP–DicB–MBP construct, the C-terminal MBP was fused to DicB through *BamH*Ι and *Xho*Ι. For constructions of different DicB truncations, various *dicB* truncation genes were inserted into pET-22b through *Nde*Ι and *Xho*Ι. Site-directed mutagenesis was used to generate the DicB mutants. For MinC and FtsZ constructs, genes encoding MinC or FtsZ were respectively inserted into pET-22b between *Nde*Ι and *Xho*Ι restriction sites. All truncations and mutants were confirmed by DNA sequencing.

### 4.3. Protein expression and purification

Bacterial BL21(DE3) cells were transformed by the plasmids described above and cultivated in LB medium. Cells were induced at the mid-log phase (OD_600_, 0.5–0.7) by 0.5 mM IPTG and harvested for disruption by sonication in buffer containing 20 mM HEPES-sodium, pH 7.5, 150 mM NaCl (buffer A). After centrifugation, the supernatant was loaded onto a HiTrap Ni-chelating column (Pharmacia). Next, beads were washed with 50 ml buffer A containing 30 mM imidazole and 300 mM imidazole was used to elute protein. Eluted samples were pooled and subjected to a size-exclusion chromatography (HiLoad Superdex16/60 S200, GE Healthcare) using an AKTA avant system (GE Healthcare). Fractions containing the desired protein were pooled, concentrated and stored at −80°C in buffer A. Proteins were analysed by SDS–PAGE and visualized by Coomassie brilliant blue (CBB) staining. Duplicated samples were transferred onto PVDF membrane and probed with a mouse monoclonal anti-His-HRP-conjugated antibody (Tianjin Sungene Biotech).

### 4.4. Pull-down assay

For MBP–DicB and MinC pull-down assay, MBP–DicB and MinC were co-expressed in BL21(DE3) cells from two separate vectors with different antibiotic resistance. The bacterial pellet was lysed by sonication in 20 mM Tris–HCl pH 7.4, 0.2 M NaCl and 1 mM EDTA (buffer B). After centrifugation, the supernatant was loaded onto an amylose column. After wash with 100 ml buffer B, 10 mM maltose was added to buffer B to elute the protein. The eluted protein was then loaded on size exclusion chromatography (HiLoad Superdex16/60 S200, GE Healthcare, 25 mM Tris–HCl, 100 mM NaCl, pH 8.0). For MBP–DicB–MBP and MinC pull-down assay, we used a different procedure in which the proteins were expressed separately. After sonication and centrifugation, individual supernatants were mixed and incubated for 1 h. Control experiments were carried out in the same manner except that MinC was expressed alone and MinC was then incubated with MBP. For MBP–DicB truncation derivatives and MinC pull-down assay, procedures were the same except that control experiments were carried out using MinC alone to confirm MinC had no non-specific binding to the amylose column. The same procedures were used for MBP–DicB (no tag) and FtsZ pull-down assay except that the supernatant was loaded onto a nickel column. Control experiments were carried out without FtsZ. Samples eluted from fast protein liquid chromatography were pooled respectively and subjected to SDS–PAGE and visualized by CBB staining.

### 4.5. Cell growth conditions

Overnight LB cultures of different DicB strains were diluted to the same OD_600_. 0.5 mM IPTG was added to cultures of each strain except for those in figure 2*b*, in which 0.1 mM IPTG was added to the cultures. The cultures were then incubated on 96-well plates with shaking at 37°C for 12 h, and cell growth was monitored by taking OD_600_ measurements hourly. During measurements, the optical path length was approximately 5 mm. Each sample was assayed in triplicate. For plate growth experiment of DicB truncation strains, procedures were the same except that the diluted strains with 0.5 mM IPTG were cultured onto LB plates at 37°C for 12 h.

### 4.6. Scanning electron microscopy

For scanning electron microscopy sample preparation, overnight cultures of strains containing different DicB derivatives or MinC/FtsZ constructs were diluted to OD_600_ 0.05 in fresh LB at 37°C. Strains were induced after 4 h with 0.5 mM IPTG and cultured for another 4 h at 37°C under 150 r.p.m. shaking. Cells were then harvested by centrifugation for 10 min at 5000 r.p.m. After washing three times with PBS (pH 7.4), cells were pre-fixed with 2.5% glutaraldehyde in 0.1 M cacodylate buffer for 30 min at 4°C. Cells were then dehydrated through a series of graded acetone solutions (10%, 30%, 50% and 70%, 10 min each) and dropped onto foil which was glued onto a metal specimen holder after the cells had dried. The SEM images of cells were obtained using a S4800 scanning electron microscope (Hitachi).

## Supplementary information

A supplementary PDF file contains cell division inhibition test of DicB fusion protein (electronic supplementary material, table S1), plasmids used in this study (electronic supplementary material, table S2), results of complementary pull-down experiments, SEM, cell growth assay, protein expression tests, plate experiments and other supporting materials (electronic supplementary material, figures S1–S9).

## Supplementary Material

Supplementary information

## Data accessibility

The datasets supporting this article have been uploaded as electronic supplementary material.
