# Supplementary material for: Characterization of DicB by partially masking its potent inhibitory activity of cell division
Source: Open Biol. 2016 Jul 27;6(7):160082. doi: 10.1098/rsob.160082 (PMC4967827; doi:10.1098/rsob.160082)
Supplement: Supplementary information [file rsob160082supp1.docx]

**Supplementary information**

**
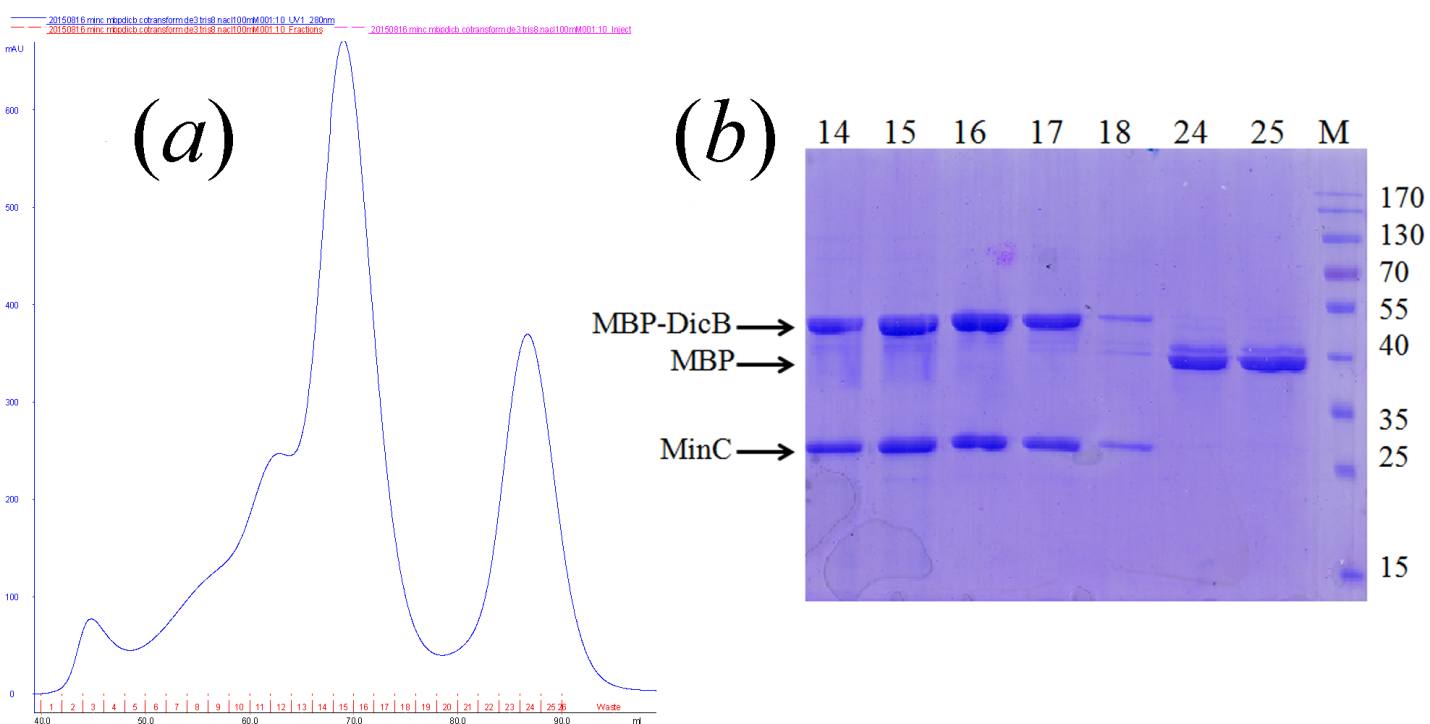
**

**Figure S1. Pull-down experiments of MinC and MBP-DicB.** (*a*) Eluted sample containing MBP-DicB and MinC was purified by gel filtration. (*b*) SDS-PAGE of gel filtration to verify the interaction between MinC and MBP-DicB. MBP-DicB readily pulled down MinC which contained no MBP tag. The observed MBP band is result of partial degradation of MBP-DicB and shows no interaction with MinC.


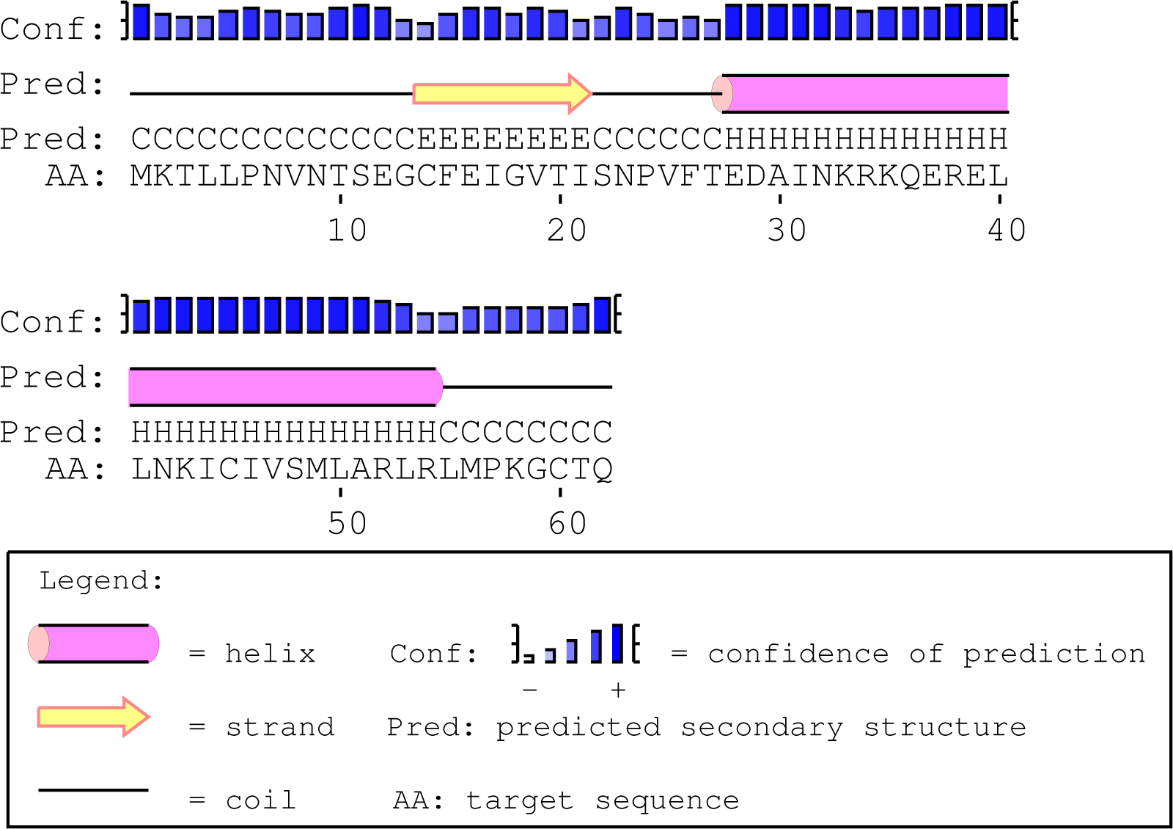


**Figure S2. Predicted secondary structure of full-length DicB (62 aa).** The secondary structure of DicB was predicted using a server from Bioinformatic Group of UCL Department of Computer Science. According to its predicted secondary structure, there is a helix from 28 aa to 54 aa exclusive of its N-terminus (1aa to ~27 aa) and C-terminus (the last 8 aa).


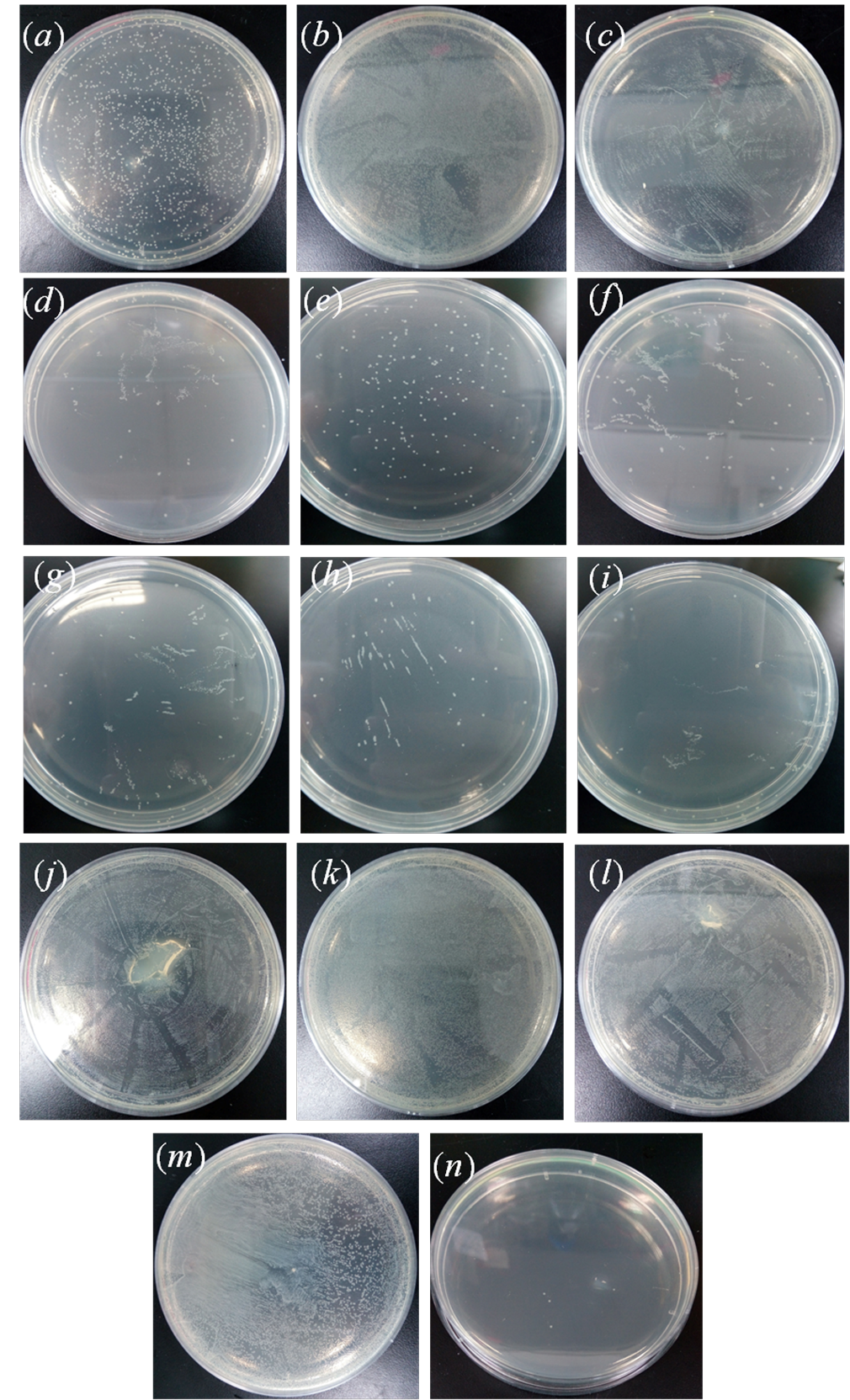


**Figure S3. LB plate growth experiment using different truncation derivatives of DicB (*a*~*l*) and MBP-DicB (*n*) expression in *E. coli* BL21(DE3).** (*a*) DicB-ΔN2; (*b*) DicB-ΔN3; (*c*) DicB-ΔN4; (*d*) DicB-ΔN5; (*e*) DicB-ΔN6; (*f*) DicB-ΔN7; (*g*) DicB-ΔN13; (*h*) DicB-ΔN26; (*i*) DicB-N26; (*j*) DicB-ΔC12; (*k*) DicB-ΔC16; (*l*) DicB-ΔC20; (*m*) *E. coli* BL21(DE3); (*n*) MBP-DicB. Colonies of DicB truncation derivatives generally were fewer than WT but more than MBP-DicB, substantiating our cell growth results in which the inhibitory activity of DicB truncations were weakened but not completely abolished.

**
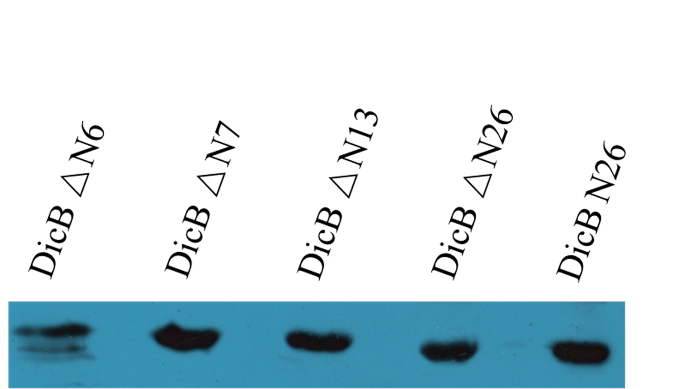
**

**Figure S4. Expression tests of DicB truncation derivatives using Western blotting.** Five representative DicB truncation derivatives were chosen to test their expression levels using a mouse monoclonal anti-His-HRP conjugated antibody. Result showed that these DicB derivatives were indeed expressed and almost in similar level.


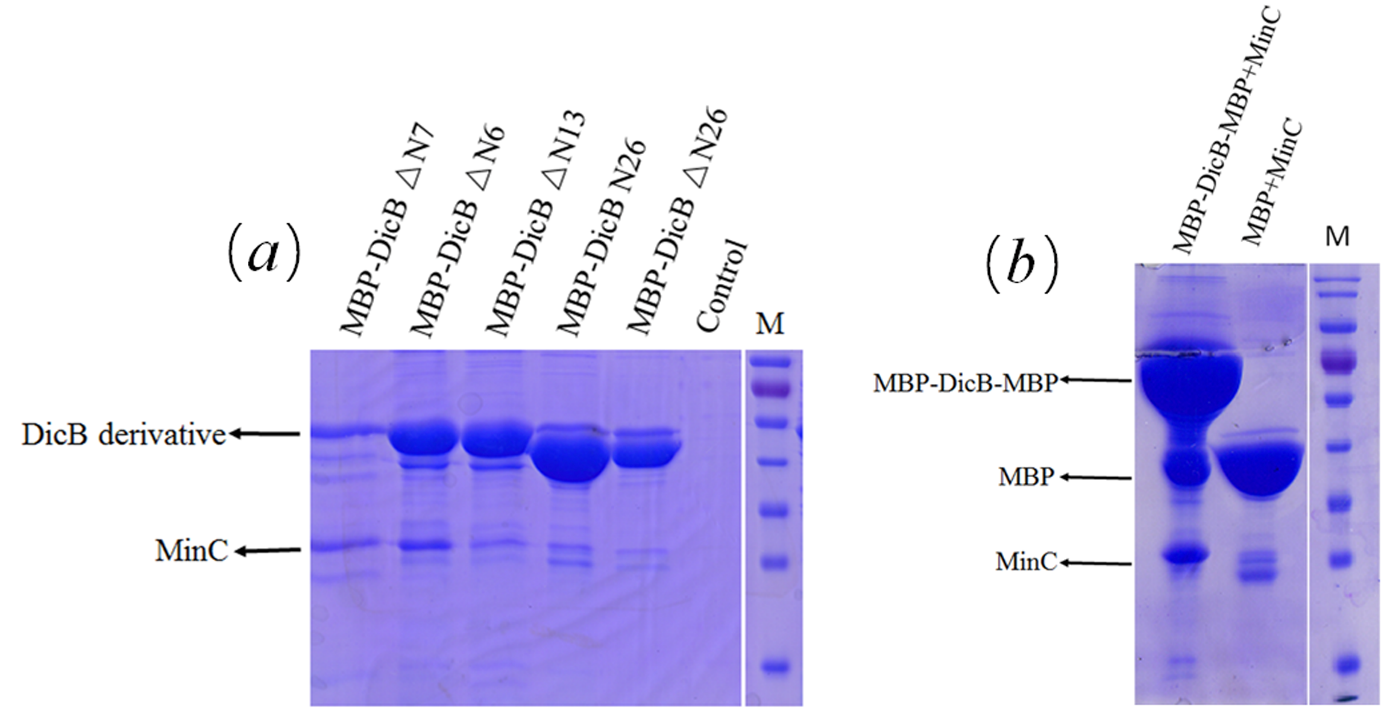


**Figure S5. Pull-down experiments between MBP-DicB derivatives and MinC.** (*a*) SDS-PAGE of pull-down experiments between MBP-DicB truncation derivatives and MinC. The control experiment was done using the same procedures without MBP-DicB truncations in order to confirm that MinC has no nonspecific binding to amylose column. MBP-DicB truncations were still able to pull down MinC which contains no MBP tag. N-terminal truncations with less residue removed (ΔN7 and ΔN6) have stronger interaction with MinC than those with more removed (ΔN13, ΔN26, N26). (*b*) SDS-PAGE of pull-down experiment between MBP-DicB-MBP and MinC. The control experiment was done in the same procedures with MBP. There exists interaction between MinC and MBP-DicB-MBP. It indicates that MBP-DicB-MBP retains interaction with MinC *in vitro* while MBP cannot interact with MinC. The white vertical line refers to the fact that MW markers are loaded on the same SDS-PAGE, but not in the adjacent lanes.





**Figure S6. Cell growth assay of MBP-DicB with FtsZ strains.** Growth curves of WT BL21(DE3) (black), MBP-DicB (red), and FtsZ (blue), in comparison with MBP-DicB and FtsZ co-expression strain (pink) under aerobic conditions at 37 °C. All experiments were completed in triplicate and performed twice. The standard error of the mean was used to calculate the error bars. For those points where experimental variations are too small, their error bars are not visible.


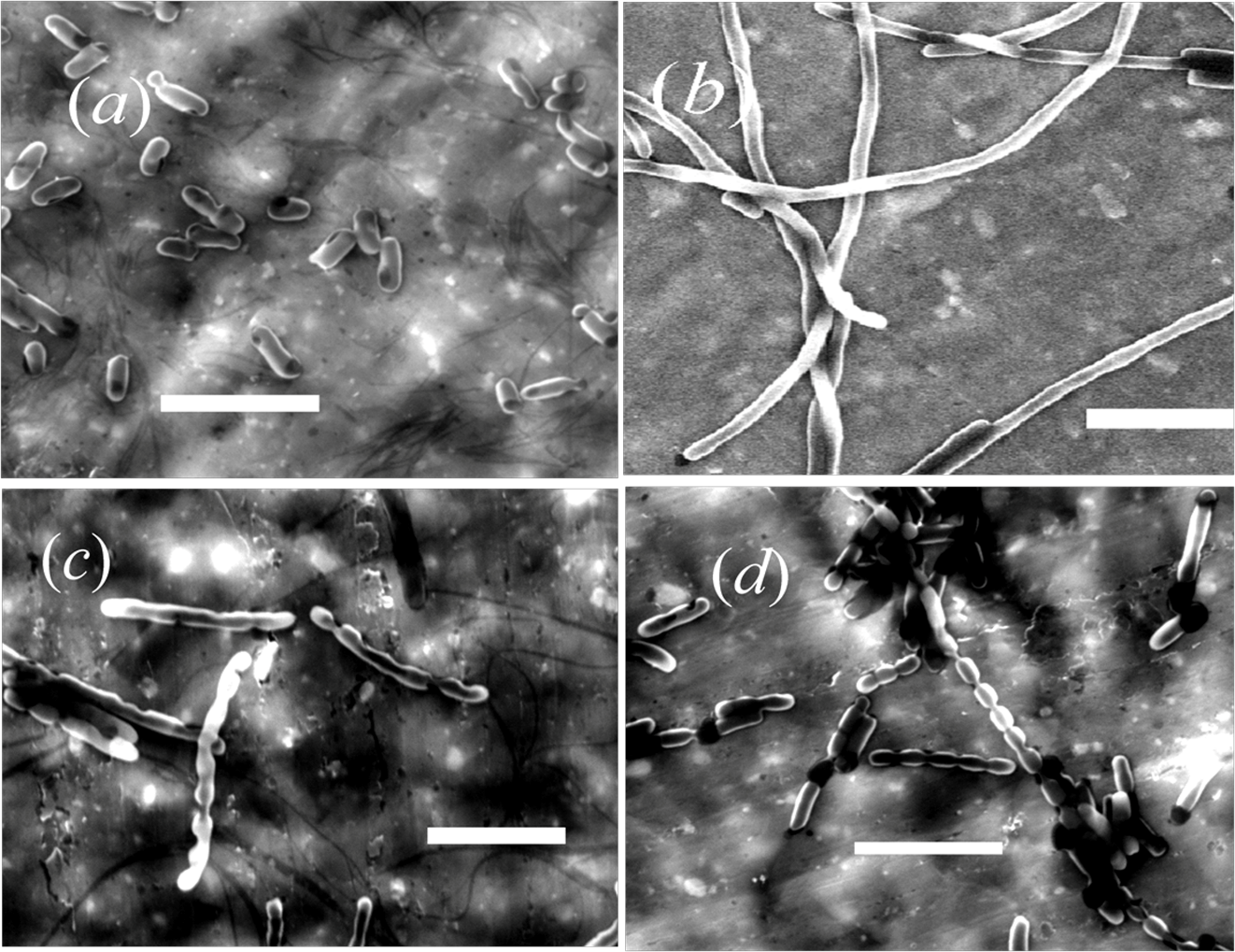


**Figure S7.** **Scanning electron micrographs of strains expressing MBP-DicB or MinC with FtsZ.** (*a*) FtsZ. (*b*) MinC. (*c*) FtsZ + MBP-DicB. (*d*) FtsZ + MinC. Compared with FtsZ strain and MBP-DicB strain (figure 3*d*), the FtsZ/MBP-DicB strain presented much shorter cell length than MBP-DicB and became strings of beads. The FtsZ/MinC strains presented similar morphology to FtsZ/MBP-DicB, which indicates that the cell division inhibitory activity of MinC/DicB is interfered by FtsZ. Scale bars represent 5 μm.


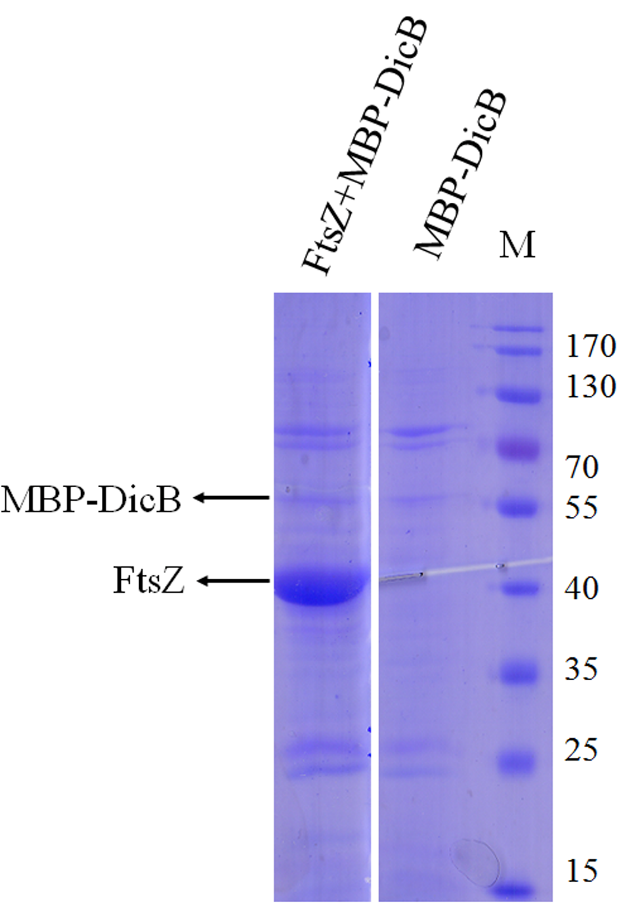


**Figure S8.** **Pull-down experiment between MBP-DicB (no-tag) and FtsZ**. The control experiment was done using the same procedures without FtsZ (His-tag) in order to confirm MBP-DicB (no-tag) has no nonspecific binding to nickel column. There was no obvious interaction between MBP-DicB and FtsZ. The white vertical line refers to the fact that FtsZ/MBP-DicB samples are loaded on the same SDS-PAGE, but not in the adjacent lanes.


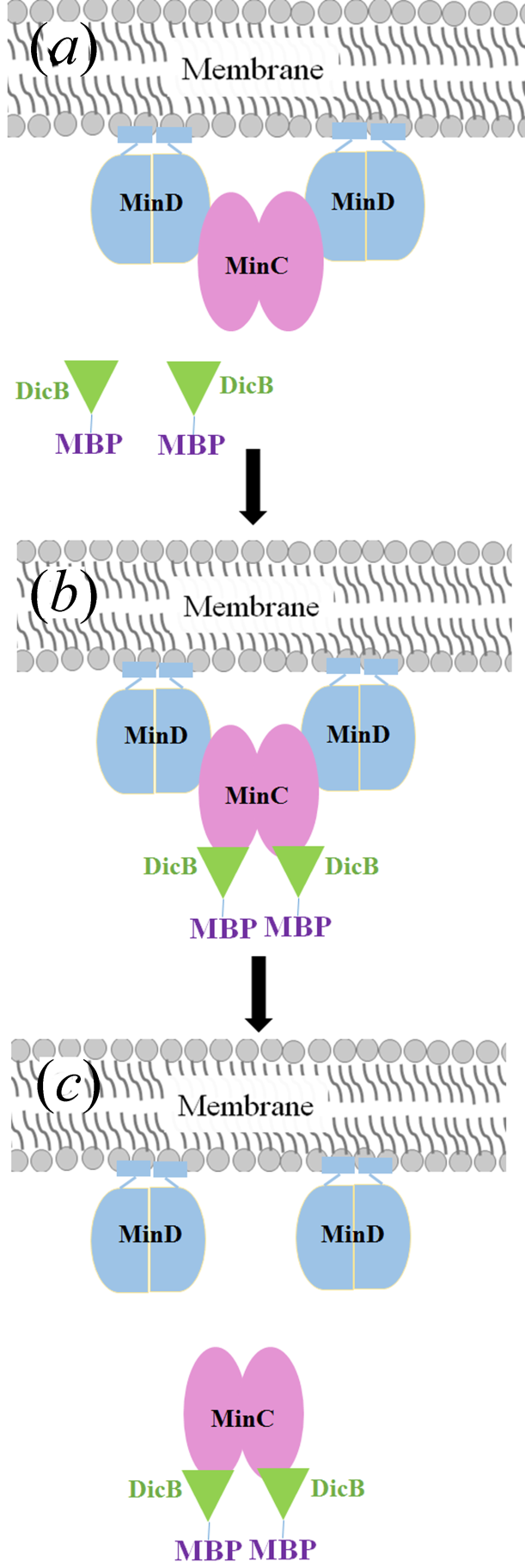


**Figure S9. Model of MBP-DicB competition with MinD to interact with MinC.** (*a*) MinD recruits MinC to the membrane and forms MinCD complex, alternating copolymer filament. (*b*) Nonetheless, once MBP-DicB reaches certain level *in vivo*, it is able to compete with MinD and interact with MinC. (*c*) As a result, MinC would dissociate from MinD and be released from the membrane, leading to the inability of cell to form proper septa at the middle of cell and abrogating cell division and growth.

**Table S1. Cell division inhibition tests of DicB fusion proteins**

| Plasmids of DicB fusion proteins | Growth on LB Plates |
| --- | --- |
| pET-28b-DicB-MBP | No |
| pET-22b-DicB-EGFP | No |
| pET-28b-MBP-DicB | Yes |
| pET-22b-EGFP-DicB | Yes |

**Table S2. Plasmids used in this study**

| Plasmids | Restriction sites | Fusion tag | Antibiotic resistance |
| --- | --- | --- | --- |
| pET-28b-MBP-DicB  pET-28b-MBP-DicB-T | *Nco*Ι/*Nde*Ι/*Xho*Ι  *Nco*Ι/*Nde*Ι/*Xho*Ι | C-His  No-tag | Kanamycin  Kanamycin |
| pET-28b-MBP-DicB-MBP | *Nco*Ι/*Nde*Ι/*BamH*Ι/*Xho*Ι | C-His | Kanamycin |
| pET-28b-DicB-MBP | *Nco*Ι/*Nde*Ι/*Xho*Ι | C-His | Kanamycin |
| pET-22b-DicB-EGFP | *Nde*Ι/*BamH*Ι/*Xho*Ι | C-His | Ampicillin |
| pET-22b-EGFP-DicB  pET-32a-DicB  pET-22b-DicB  pET-28b-DicB  pET-22b-DicB-derivative*^a^*  pET-22b-MinC  pET-22b-FtsZ | *Nde*Ι/*BamH*Ι/*Xho*Ι  *Nco*Ι/*Xho*Ι  *Nde*Ι/*Xho*Ι  *Nde*Ι/*Xho*Ι  *Nde*Ι/*Xho*Ι  *Nde*Ι/*Xho*Ι  *Nde*Ι/*Xho*Ι | C-His  N-His  C-His  N-His  C-His  C-His  C-His | Ampicillin  Ampicillin  Ampicillin  Kanamycin  Ampicillin  Ampicillin  Ampicillin |

*^a^*pET-22b-DicB-derivative refers to the constructs of different DicB truncations and mutants listed in table 1.
